# Precise engineering of cetuximab encapsulated gadollium nanoassemblies: *in vitro* ultrasound diagnosis and *in vivo* thyroid cancer therapy

**DOI:** 10.1080/10717544.2021.1889721

**Published:** 2021-03-12

**Authors:** Ming Qi, Shufeng Gao, Sihui Nie, Ke Wang, Lingling Guo

**Affiliations:** a Ultrasound Department, The First Affiliated Hospital of Jinzhou Medical University, Jinzhou, China; b Functional Division, Jinzhou Yixian People’s Hospital, Jinzhou, China

**Keywords:** Gadolium nanoassembly, thyroid cancer, apoptosis, ultrasound diagnosis, in vivo antitumor efficacy

## Abstract

We report the formulation of nanoassemblies (NAs) comprising C225 conjugates Gd-PFH-NAs (C-Gd-PFH-NAs) for low-intensity focused ultrasound diagnosis ablation of thyroid cancer. C-Gd-PFH-NAs showed excellent stability in water, phosphate-buffered saline (PBS), and 20% rat serum. Transmission electron microscopy (TEM) images also revealed the effective construction of C-Gd-PFH-NAs as common spherical assemblies. The incubation of C625 thyroid carcinoma with C-Gd-PFH-NAs triggers apoptosis, as confirmed by flow cytometry analysis. The C-Gd-PFH-NAs exhibited antitumor efficacy in human thyroid carcinoma xenografts, where histopathological results further confirmed these outcomes. Furthermore, we were able to use low-intensity focused ultrasound diagnosis imaging (LIFUS) to examine the efficiency of C-Gd-PFH-NAs in thyroid carcinoma *in vivo*. These findings clearly show that the use of LIFUS agents with high performance imaging in different therapeutic settings will have extensive potential for future biomedical applications.

## 1. Introduction

Anaplastic thyroid carcinoma (ATC) is one of the most malignant carcinomas, though it is comparatively rare, characterized by fast proliferation, neck invasion, and remote metastasis (Zhu et al., 2018; Zhong et al., 2019; Zhou et al., 2019; Wang et al., 2020). The severe prognosis that accompanies ATC is due to the rapid progression of tumors before diagnosis. Current treatment is based on different combinations of chemotherapy and exterior beam radiation, which have been unsuccessful in improving survival, where average survival rates are 4–6 months with less than 20% survival in 12 months (Tokranova et al., 2013; Liu et al., 2018; Yang et al., 2020). Therefore, new theranostic approaches for initial detection and efficient ATC treatment are needed (Iwashina et al., 2006; Kang et al., 2019; Ho et al., 2020).

Recent studies have extensively explored systems that couple triggerable drug-charged nanocarriers with multiple inner or external stimuli, such as pH, temperature, ultrasound, laser, and microwave radiation, to enable controlled drug release for personalized treatment. These systems have excellent potential for delivering enhanced anticancer treatment, while also decreasing systemic toxicity (Cutignano et al., 2017; Pan et al., 2018; Wang et al., 2020). Low-intensity concentrated ultrasound (LIFUS) has been exhaustively researched for tumor treatment with ultrasound imaging analysis as one of the probable exterior activators, as it is noninvasive and displays significant tissue-penetrating capacity. In particular, it can significantly increase the efficacy of chemotherapy, avoiding harm to nearby cells and reducing adverse side effects (Paolino et al., 2008). However, the discharge of LIFUS-triggered drugs from nanocarriers for improved tumor therapy is still unsatisfactory, largely due to the comparatively lower accumulation efficiency of nanoparticle-charged nanotransporters at tumor sites. Numerous nanotransporters have been extensively characterized for enhanced aggregation of a large number of tumors while minimizing side effects (Paolino et al., 2008; Balbín et al., 2014; Zheng et al., 2019).

Several reports have shown that overexpression of the epidermal growth factor receptor (EGFR) is strongly associated with tumor progression, migration, and invasion. EGFR is common in ATC patients (Parker et al., 2016) and antibodies or small molecules based on EGFR immunotherapy can significantly increase the therapeutic effect against this cancer. A human murine chimeric EGFR-targeted monoclonal antibody called cetuximab has higher specificity for the extracellular domain of human EGFR and inhibits epidermal growth factor signaling in cells by delaying usual receptor function (Lee et al., 2010; Zhou et al., 2015; Kong et al., 2019). The US Food and Drug Administration approved preclinical treatments using cetuximab for EGFR-expressing cancer tumors, neck and head carcinomas, and colorectal carcinomas. This C225 antibody might be a suitable objective for modifying the structure of nanocarriers to improve the therapeutic outcome in ATCs. Remarkably, some researchers have revealed that for a wide spectrum of cancers, the blend of C225 with CPT-11 equivalents such as Gd-PFH-NAs has significant synergetic antitumor effects (Heskamp et al., 2014; Zeng et al., 2014; Hu et al., 2016). Hence, Gd-PFH-NAs in combination with C225 could also enhance ATC diagnostics. However, owing to the reduced vascular dispersal of C225 and the hydrophobicity of Gd-PFH-NAs, the NAs penetration of the growth and their quantity in the tumor area were inherently imperfect, showing greatly debilitated anticancer efficacy. In contrast, these problems can be minimized by incorporating Gd-PFH-NAs and C225 into one nanotransporter to attain a combination chemotherapy while simultaneously providing targeting capability to the nanocarriers (Sung et al., 2006; García-Fernández et al., 2017; Kim et al., 2017, 2021).

Furthermore, medical imaging is essential to early diagnosis and monitoring of tumor progression. Numerous researchers have proposed that LIFUS has the potential to achieve concurrent US medication transfer, meeting the present need for initial treatment and ATC therapy (Suriano et al., 2006; Kim et al., 2016, 2019). The large and variable dimensions of microbubbles are not appropriate for drug delivery purposes, though they demonstrate outstanding agents for imaging. Realization of the tumor theranostic strategy by conservative US agents may require intensively studied phase-changing NAs that could be activated via LIFUS. Phase-changing NAs provide important benefits in tumor theranostics, facilitating tumor ultrasound and ultrasound-triggered drug release (Naso et al., 2014; Wang et al., 2014; Sathiya Kamatchi et al., 2020). This new strategy offers potential development of malignancy treatments and addresses the present theranostic needs in ATC significantly.

**Figure 1.**
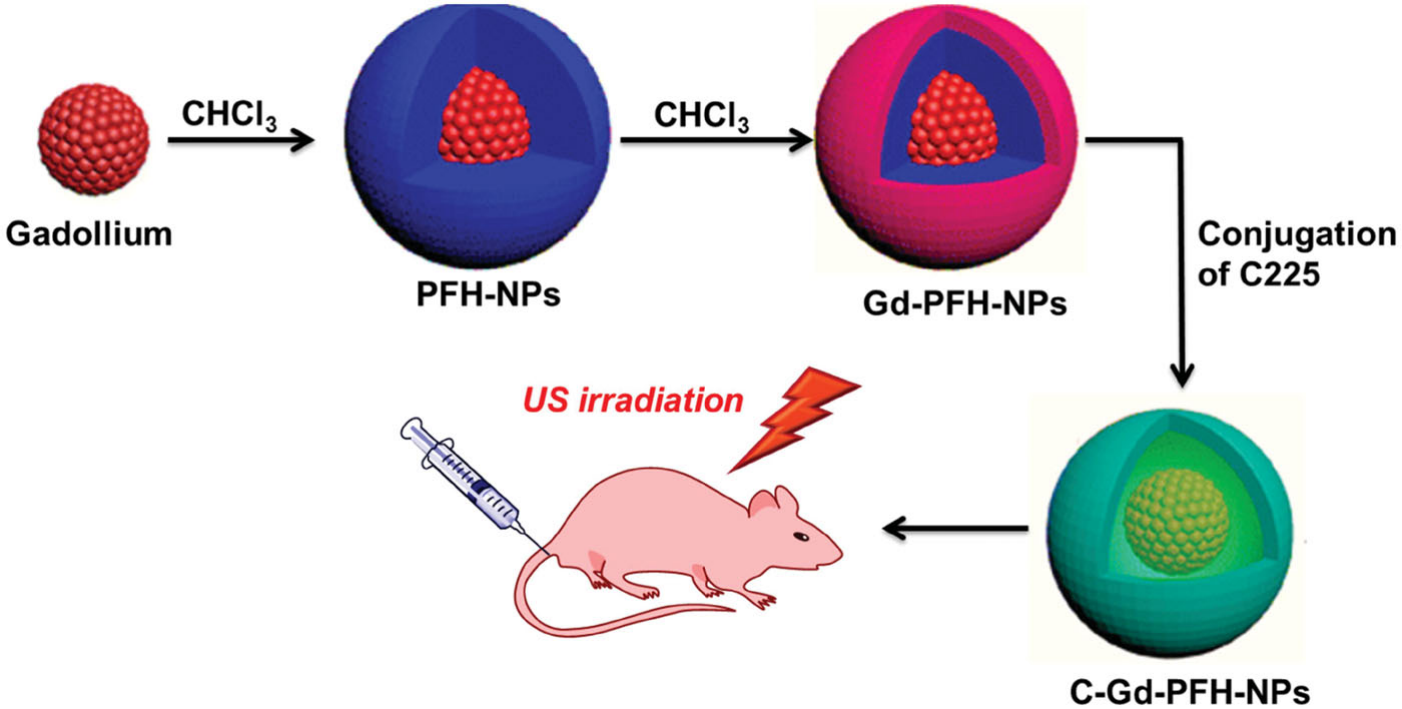
Schematic illustration of the microstructure of C-Gd-PFH-NPs and the phase-transformation process by means of LIFUS ultrasound irradiation. Meanwhile, a schematic of LIFUS ablation principles.

The objective of this work was to construct the modification of C225 nanocarrier to exactly prevent ATC that might accrue in cancer cells, in addition to the enhanced permeability and retention (EPR) effect, through the great tumor homing belongings of C225. The Gd-PFH-NAs payload could be released and LIFUS-triggered synergistic chemotherapy with C225 may perhaps suggestively make best use of therapeutic efficacy, improve USI and diminish the side effects of chemotherapy. As shown in Figure 1, due tothe tremendous biodegradability and biocompatibility, we used a PHF (perfluorohexane) core as the shell structure of the nanocarrier. We then synthesized phase-changing NAs with perfluorohexane liquid (PHF, 29 °C boiling point). Meanwhile, Gd-PFH-NAs were burdened into the nanoparticles at the similar period of time as C225 was conjugated on surface of Gd nanoparticles afford (C-Gd-PFH-NAs) C225-conjugated Gd-PFH-NAs-charged phase transformation. To our knowledge, this is the first work of a LIFUS-mediated C225 modified nanosyste that assimilates tumor targeted both US imagery and US activated drug conveyance to ATC.

## 2. Experimental section

### 2.1. Materials

Perfluoropentane (PFP, boiling point of 29 °C), *N*-(3-dimethyl-aminopropyl)-*N*′-ethylcarbodiimide hydrochloride (EDC), and fluorescent dyes (1,1′-dioctadecyl-3,3,3′,3′ tetramethylindocarbocyanine perchlorate (DiI), *N*-Hydroxysuccinimide (NHS), Lysotracker and 4′,6-diamidino-2-phenylindole (DAPI)) were purchased from Sigma-Aldrich (St. Louis, MO, USA). Cetuximab (C225, Erbitux) was purchased from Merck KGaA Co. (Frankfurter, Germany). FITC, Tween 80, RPMI-1640 medium (1640) and fetal bovine serum (FBS) was purchased from Abcam Co. (Shanghai, China). Cell Counting Kit-8 (CCK-8) was obtained from Dojindo Molecular Technology (Shanghai, China). Trichloromethane and dimethyl sulfoxide (DMSO) were purchased from Chongqing Chuandong Chemicals (Chongqing, China). All other reagents used in this work were of analytical grade and were used as received.

### 2.2. Synthesis of C-Gd-PFH-NAs

Gadollium (Gd) and perfluorohexane (PFH) nanoparticles (Gd-PFH-NAs) were fabricated by a film hydration method coupled with a double emulsion method. At first, 100 mg of lipids compounds including dipalmitoylphosphatidylcholine (DPPC), 1,2-distearoyl-sn-glycero-3-phosphoethanolamine-N-[folate(polyethylene glycol)-2000] (DSPE–PEG (2000)-folate), 1,2-Dipalmitoyl-sn-glycero-3-phosphoglycerol (DPPG), and cholesterol (CH) with mass ratios of 5:2:1.5:1.5 were dissolved in 10 mL aqueous solution until clear. Then, 100 mL of Au (10 mg/mL) was added to the aqueous solution. The mixture was transferred into the rotary evaporator (Yarong Inc., Shanghai, China) and rotated at 55 rpm and 45 °C to clear the aqueous solution. The thin lipid film formed was rehydrated with 5 mL PBS to generate a brown suspension. The suspension was then dispersed with a high-speed homogenizer (FJ300-SH, Shanghai, China) for 5 m after drop-by-drop addition of 500 µL PFH. The secondary emulsion was performed by means of an ultrasonic oscillator (SONICS & MATERIALS Inc., USA) for 5 m in an icecold environment (0 °C). Finally, the mixture of nanoemulsions was harvested and centrifuged (Eppendorf, Germany) at 4500 rpm for 5 m and washed with deionized water for three times to sweep away dissociative Au and PFH. The final emulsion was collected and stored at 4 °C for further use. Fluorescent nanoemulsions were obtained according to the above procedure except that the DiI was blended in the lipids solution.

### 2.3. C225 conjugation

Conjugation of C225 to the Gd-PFH-NAs loaded nanoparticles was performed using carbodiimide chemistry. Briefly, the prepared Gd-PFH-NAs were dissolved in 5 mL of MES buffer solution (0.1 M, pH 5.5) together with a mixture of 3 mg of EDC and 10 mg of NHS, and then incubated vigorously for a period of 1 h on a gentle shaker. The resulting solution was centrifuged and washed three times with PBS to remove unreacted 1-ethyl-3-(3-dimethylaminopropyl)carbodiimide (EDC) and 10 mg of *N*-hydroxysuccinimide (NHS). Then, the sediment was redissolved in 5 mL of 2-(*N*-morpholino)ethanesulfonic acid (MES) buffer solution (0.1 M, pH 8.0). Next, excess C225 was dropped into the above solution and stirred on a gentle shaker for another 2 h. After the reaction was completed, Gd-PFH-NAs with C225 conjugation (C-Gd-PFH-NAs) were obtained by centrifugation, washed thrice with PBS again to remove unconjugated C225 and preserved at 4 °C before use. All the aforementioned procedures were carried out in an ice bath.

### 2.4. Characterization of C-Gd-PFH-NAs

Optical microscopy (CKX41; Olympus, Tokyo, Japan) and confocal laser scanning microscopy (CLSM) (Nikon A1, Tokyo, Japan) were employed to observe the morphology and particle distribution of Gd-PFH-NAs and C-Gd-PFH-NAs. A dynamic light scattering analyzer (DLS) (Malvern Instruments, Malvern, UK) was used to determine the mean particle size and polydispersity index (PDI) NAs. Morphological characterization of NAs was carried out by transmission electron microscopy (TEM; H-7500; Hitachi, Tokyo, Japan). The mean particle size of the nanoparticles was determined by DLS within 7 days to evaluate the stability of the Gd-PFH-NAs and C-Gd-PFH-NAs.

### 2.5. Cell culture and nude mice

The Cell Bank of the Chinese Academy of Sciences (Shanghai, China) acquired a human anaplastic thyroid carcinoma line (C643). The cells were grown in medium RPMI-1640 containing 10% FBS and 1% penicillin-streptomycin at 37 °C in humidified air with 5% CO_2_. At the Laboratory Animal Center of Department of Ultrasound, Harbin Medical University Cancer Hospital (Harbin, China), BALB/C Female both mice and nude mice (balancing about 19 g, 25 days) were bought then raised. All animals on our studies were collected from the Harbin Medical University Cancer Hospital Laboratory Animal Center and retained in accordance with rules authorized by the Harbin Medical University’s Animal Ethics Committee (Harbin, China). Furthermore, all animal experimental activities were strictly in line with the policy of the Harbin Medical University’s Institutional Animal Care and Use Committee (IACUC), and this study was endorsed by the IACUC.

In order to start an ATC model in nude mice, C643 cells were collected, splashed thrice with the FBS-free medium of RPMI-1640, and subcutaneously inoculated into each mouse’s left flank (3 **×** 10^7^ C643 cells in 150 μL FBS-free medium of RPMI-1640 for each mouse. A Vernier caliper was used to measure the length and width of the tumor and the tumor quantity was considered by the calculation: volume-(length as width**×** 2)/2.

### 2.6. *In vitro* analysis

#### 2.6.1. *In vitro* intracellular uptake C-Gd-PFH-NAs

In cultivation dishes, seeded the C643 cells for CLSM at a mass of 1 × 10^6^ cell mL/dish, grown at 37 °C in moistened air comprising 5% CO_2_. The cells were spilt into four groups after 24 h of culture: C-Gd-PFH-NAs were handled respectively with 10 min and 15 min Dil-labeled C-Gd-PFH-NAs (1 mg/mL), and after blocking the cells were washed three times with phosphate-buffered saline (PBS). Then, Dil-labeled C-Gd-PFH-NAs (1 mg/mL) incubated the cells. The cells were washed with PBS three times after 2 h incubation with nanoparticles, fixed with 4% paraformaldehyde (200 μL) for 15 minutes, and then gestated by 6-diamidino-2-phenylindole (DAPI) (10 μg/mL, 200 μL) for 20 min. Lastly, CLSM pictured the dishes (Nair & Sharma, 2012; Nabi-Meibodi et al., 2013; Kamiya & Takeuchi, 2017).

#### 2.6.2. *In vitro* cytotoxicity assay

The CCK-8 assay assessed the cell viability. C643 cells were seeded into 96-well plates (1 × 10^3^ cells per well, 100 μL). After 24 h incubation to assess the cell viability Gd-PFH-NAs and C-Gd-PFH-NAs treated at levels of 10, 5, 2.5, 1.25, 0.625 and 0.312 µM for 24 h. Gd-PFH-NAs and C-Gd-PFH-NAs cells were incubated for 24 h. The positive control used as the untreated C643 cells. The *in vitro* cytoxicicty assay performed and the calculated made by the company manufactures guidelines.

#### 2.6.3. Apoptosis examinations

The cells were seeded (4 × 10^6^ C643 cells per well, 1.5 mL) into a 6-well dish and grown at 37 °C in a humidified incubator with 5% CO_2_ for 24 h. The IC_50_ concentration used by Gd-PFH-NAs and C-Gd-PFH-NAs. The cell apoptosis assay grouping technique was in accordance with the cell viability assay group. After administering IC_50_ concentration of the formulations of Gd-PFH-NAs and C-Gd-PFH-NAs was implemented 2 h later (Mohan et al., 2018; Mohamed Subarkhan et al., 2019; Balaji et al., 2020).

#### 2.6.4. Cell cycle arrest examinations

The cells were seeded (4 × 10^6^ C643 cells per well, 1.5 mL) into a 6-well dish and grown at 37 °C in a humidified incubator with 5% CO_2_ for 24 h. The IC_50_ concentration used by Gd-PFH-NAs and C-Gd-PFH-NAs. The cells were gathered and analyzed in the PI-stained cells after 24 h of culture, and the percentages of the cells in the G0/G1, S phase, and G2/M phases were evaluated (Mohamed Subarkhan et al., 2016; Subarkhan & Ramesh, 2016; Mohamed Kasim et al., 2018).

#### 2.6.5. *In vitro* fluorescence imaging in xenografts tumor

A continuous dosage of DiR labeled Gd-PFH-NAs and C-Gd-PFH-NAs (2 mg/mL, 200 μL) was given to C643 tumor-bearing mice. With 1% pentobarbital, all mice were totally narcotized and fluorescence pictures were acquired before injection and 3 h, 6 h and 24 h post-injection. A vivid fluorescence imaging for tiny animals evaluated the fluorescence intensity changes in the tumor areas *in vivo*. For *ex vivo* fluorescence imaging, the significant organs and tumor of one mouse were gathered. In addition, Dil-labeled Gd-PFH-NAs and C-Gd-PFH-NAs (2.5 mg per mL, 150 μL) were injected through the intravenous of C643 tumor-bearing mice were injected 6 h after injection. At the predetermined post-injection moment, tumor matters and significant tissues were gathered, segmented, and ice-covered. DAPI dyeing was conducted in the dark for 5 min after fastening with 4% paraformaldehyde. The biodistribution of Dil-labeled Gd-PFH-NAs and C-Gd-PFH-NAs was monitored by CLSM (Kabeer et al., 2019; Yalcin et al., 2020; Zhang et al., 2020).

### 2.7. Therapeutic efficacy *in vivo*

When the subcutaneous tumor reaches 100 mm^3^ in volume, an antitumour assay was conducted on xenografts of mice carrying anaplastic thyroid cancer. The tumor-bearing mice were arbitrarily split into 3 communities (n-5 per unit): control group (saline) and free Gd-PFH-NAs and C-Gd-PFH-NAs were administered. 200 µL of the blend was injected with the same dose of Gd-PFH-NAs and C-Gd-PFH-NAs (1 mg/kg) through the tail vein in a 1% saline solution were determined 6 h after injection with the US agent filling the investigation with the tumor superficial. Afterward the inoculations of C643 cells, 5 consecutive treatments were performed each 72 h starting on day 20 and ending on day 37. Each mouse’s tumor dimensions and weight was recovered every three days, and changes in tumor volume were examined from the relative tumor dimensions V/V_0_ (V_0_: initial volume prior to treatment), and tumor growth curves were drawn at the same time. On day 21 days, all mice were euthanized and dissected and weighed the tumor masses. In addition, studies in histology and immunohistochemistry were conducted. Sections of the tissue were stained with histophalogy.

### 2.8. Statistical assay

Each experiment was repeated at least three times. The mean ± standard deviation of all the data was analyzed in the tables and figures. One-way ANOVA was utilized for the inter-group comparison, and the bilateral paired *t*-test and the least significant difference (LSD) test were utilized for statistical evaluation. *p* values <.05 were considered statistically significant (*p < .05, **p < .01, ***p < .001, ****p < .0001).

## 3. Results and discussion

### 3.1. C-Gd-PFH-NAs characterization

**Figure 2.**
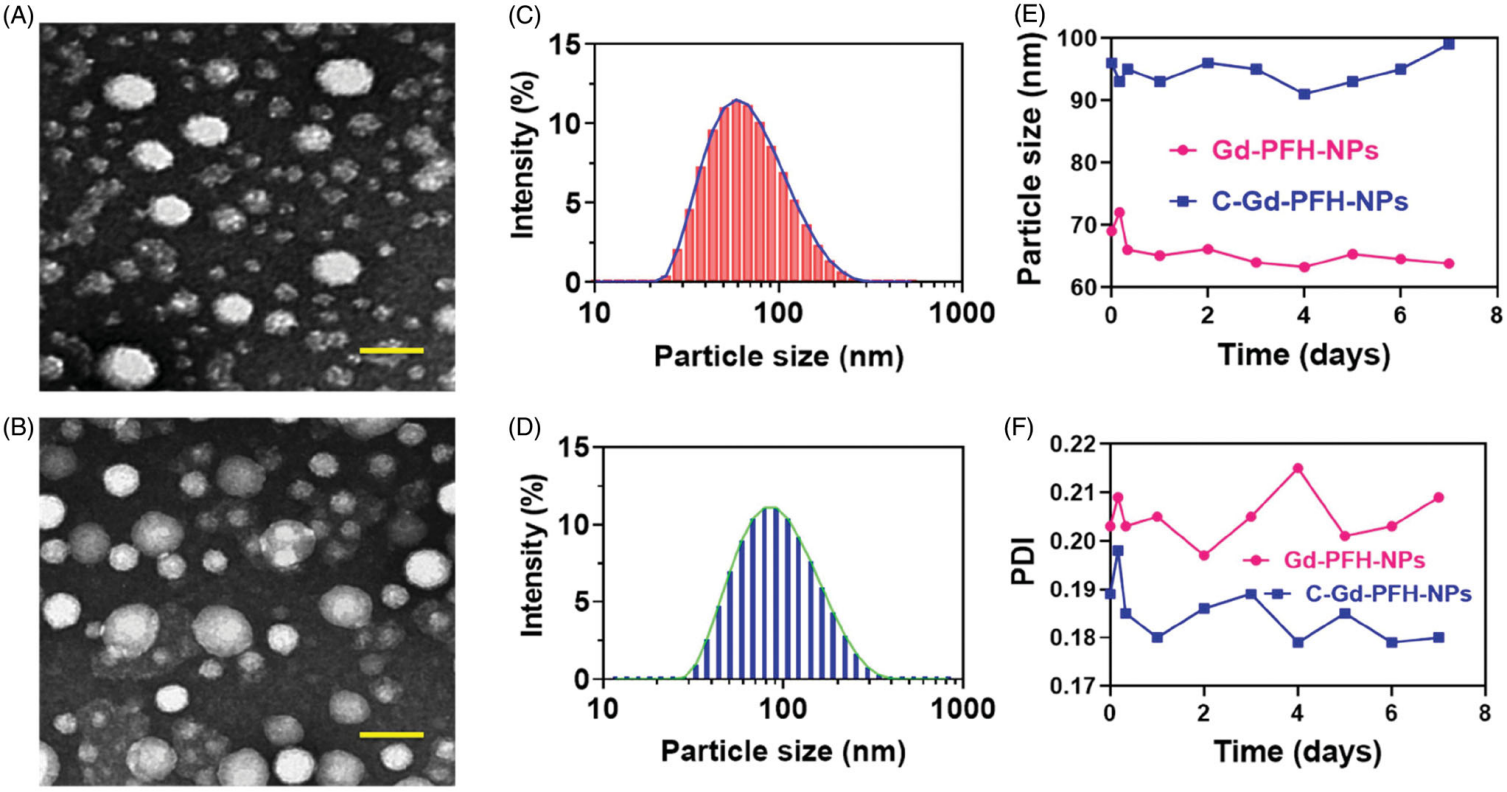
A) TEM image of Gd-PFH-NPs and B) C-Gd-PFH-NPs. Scale bars, 100 nm (B and D) DLS image of Gd-PFH-NPs and C-Gd-PFH-NPs. (E and F) Stability of Gd-PFH-NPs and C-Gd-PFH-NPs. in water with PBS at 37 °C.

With Gd-PFH-NAs and C-Gd-PFH-NAs in hand, we examined these compounds using TEM analysis (Figure 2(A)). We next tested whether they were able to recapitulate the expected self-assembly behavior in aqueous solutions. For this purpose, we dissolved the C-Gd-PFH-NA prodrugs in dimethyl sulfoxide (DMSO) (10 mg/mL) and then rapidly injected them into deionized (DI) water under ultrasonication. This procedure allowed us to verify that the solution was transparent and slightly bluish. Electron microscopy revealed that the drug molecules self-assembled to form a spherical nanoparticle structure and DLS showed a single peak distribution of the nanoparticles. The average hydrodynamic diameter (intensity) of compound 1 was approximately 107.1 nm, while the diameter of compound 2 was approximately 108.0 nm (Figure 2(B)). However, adhesion is observed between nanoparticles formed upon self-assembly with simple small-molecule drugs. Therefore, we generate miscible Gd nanoparticles by combining the prodrug with the appropriate amount of C225 molecules. These nanoassemblies are formed and have been widely used for *in vivo* drug delivery in order to solve the problem of adhesion and optimize cancer-specific drug delivery. Next, we measured the stability of C-Gd-PFH-NAs in various solvents, such as water, PBS, 20% rat serum, and tween-20, and found a significantly stable size in each (Figure 2(C)). Taken together, these results suggest that although C-Gd-PFH-NAs can self-assemble to form nanoparticles, they may not be sufficiently stable. Therefore, C225 nanoparticles loaded with Au-PFH were investigated further to evaluate their anticancer efficacy *in vitro*.

### 3.2. *In vitro* intracellular uptake

**Figure 3.**
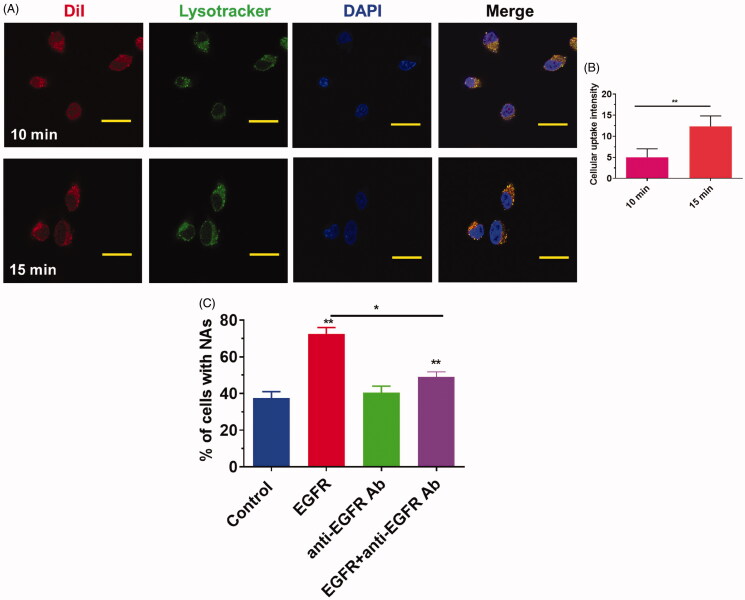
A) Cellular uptake of C-Gd-PFH-NPs with 10 min and 15 minutes interval. B) Cellular uptake flurosence intensity ratio of C-Gd-PFH-NPs with 10 min and 15 minutes interval. C) EGF enhanced C-Gd-PFH-NPs cellular uptake efficiency by C643 cells. C643 cells were incubated with 100 ng/mL of EGF (second bar), 20 ng/mL of anti-EGFR antibody, or 100 ng/mL of EGF and 20 ng/mL of anti-EGFR antibody at 37 °C for 24 h. The cellular uptake efficiency of NPs was normalized to that of untreated control cells. *p ≤ .05, **p ≤ .01 compared to each normalized control.

As illustrated in Figure 3(A,B), a much stronger red fluorescence was derived from cells transfected with Dil-labeled C-Gd-PFH-NAs for 10 and 15 minutes. Furthermore, a stronger red fluorescence was noted in cells after exposure to the C-Gd-PFH-NAs group. These findings revealed that the elevated tumor-homing characteristics of C225 allowed the C-Gd-PFH-NAs to fix tightly to the C643 cells, facilitating intracellular uptake considerably. In the resentment group, C-Gd-PFH-NAs lost the capacity to target the C643 cells because surplus free C225 led to congestion, resulting in low levels of C-Gd-PFH-NAs around the cells and demonstrating that the EGFR-mediated targeting was effective in C-Gd-PFH-NAs. The effect of EGF on the cellular uptake of C-Gd-PFH-NAs by C643 cells was examined and the results are shown in Figure 3(C). The percentage of control cells containing C-Gd-PFH-NAs was approximately 30%, whereas the cellular uptake ratio of C-Gd-PFH-NAs by cells treated with 100 ng/mL of EGF increased to approximately 70%. EGF enhanced cellular uptake of C-Gd-PFH-NAs by dose dependent manor (data not shown), and 100 ng/mL of EGF showed higher enhancing than lower concentration of EGF. We confirmed the increase in cellular uptake by EGF and EGFR by adding anti-EGFR antibody, which blocks the binding of EGF to EGFR. We first tested the effect of anti-EGFR antibody (20 ng/mL) alone or in combination with EGF (100 ng/mL). Treatment with anti-EGFR antibody (20 ng/mL) alone had no significant effect on the uptake ratio of C-Gd-PFH-NAs by C643 cells, whereas treatment with a combination of EGF and anti-EGFR antibody significantly decreased the uptake ratio. Anti-EGFR antibody blocked cellular uptake by dose dependent manor (data not shown), and 20 ng/mL of anti-EGFR antibody showed higher blocking effects than lower concentration of antibody. The results suggested that the EGF-EGFR complex participates in cellular uptake triggered by an increase in EGF.

### 3.3. *In vitro* cytotoxicity assay

**Figure 4.**
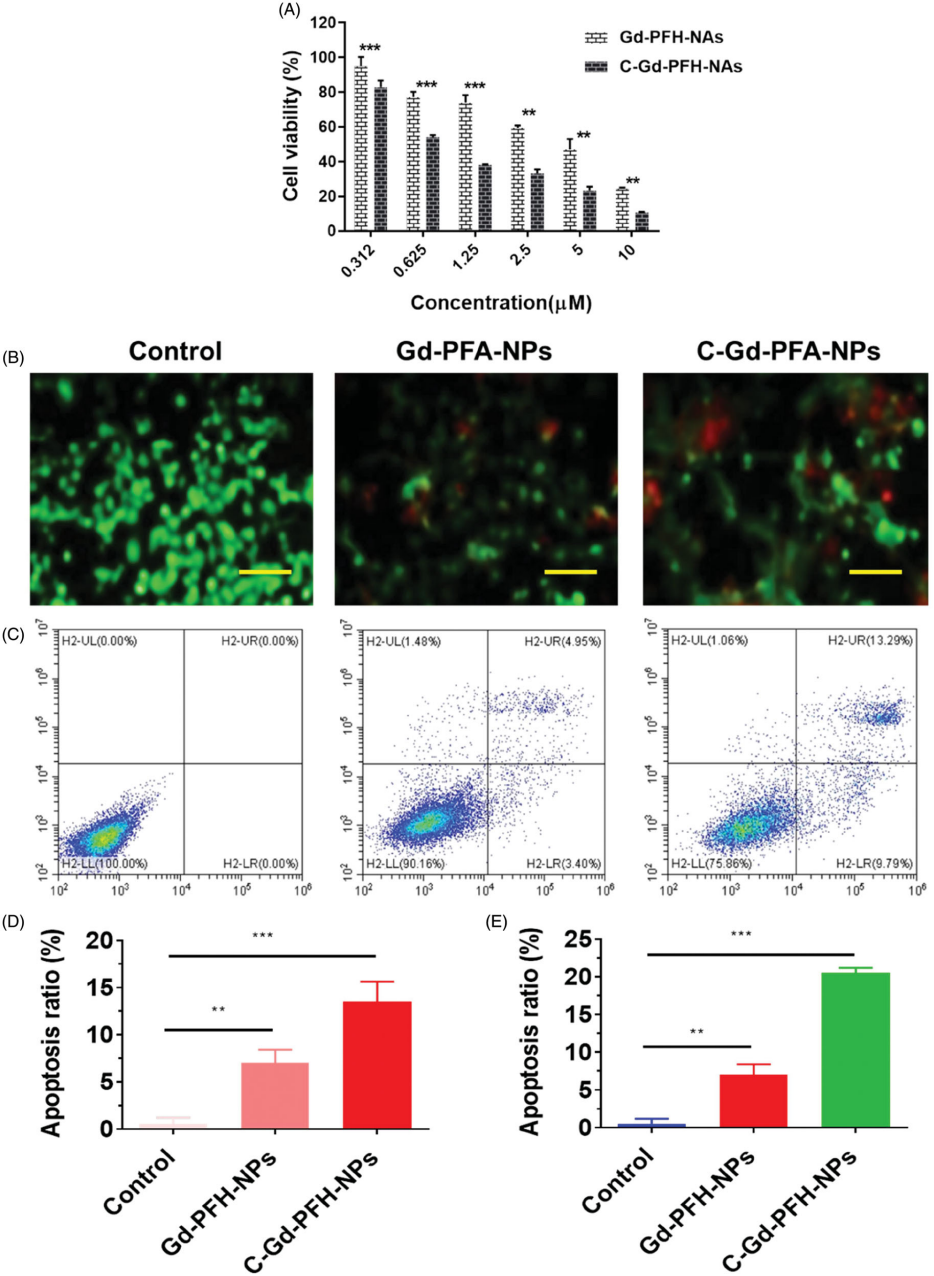
A) Dual AO/EB staining assay for examining Gd-PFH-NPs and C-Gd-PFH-NPs -induced cell death in C643 cells. The cells were treated with Gd-PFH-NPs and C-Gd-PFH-NPs at 2.5 µM concentration for 24 h. B) Quantification of apoptosis ratio. The cells were quantified by image J software. C) Apoptotic analysis of C643 cells using flow cytometry. The cells were treated with Gd-PFH-NPs and C-Gd-PFH-NPs at 2.5 µM concentration for 24 h and then stained with FITC annexin V/PI for flow cytometry analysis. D) Apoptosis ratio of C643 cells. ***p ≤ .005, **p ≤ .01 compared to each normalized control.

The CCK-8 assay was used to assess cell viability with different NP formulations at multiple concentrations, showing a dose-dependent model. As illustrated in Figure 4(A), the cell viability of nanoparticles in the analyzed dose range was more than 80%, with concentrations ranging from 0.312 to 0.625 μM. The comparatively small (insignificant) loss of viability suggested that the elevated biocompatibility of these phase-changing nanoparticles was appropriate for *in vivo* applications. As expected, Gd-PFH-NAs and C-Gd-PFH-NAs cell viabilities decreased considerably as the levels of C-Gd-PFH-NAs increased. In particular, the viability of cells treated with C-Gd-PFH-NAs was lower than Gd-PFH-NAs at the same concentration, implying that the mixture in C-Gd-PFH-NAs could boost cytotoxicity synergistically. The remarkably improved cytotoxicity of C-Gd-PFH-NAs may be due to the increased cell membrane permeability, caused by the cavitation effect, and the improved C-Gd-PFH-NAs at the target site, which significantly increased the inhibitory effect of C-Gd-PFH-NAs on cell development.

### 3.4. Apoptosis in C643 cancer cells

Apoptosis may be reckoned as an important obstacle for a damaged cell to become malignant tumors. Since the complexes promote apoptosis induction in cancer cells, flow cytometry using annexin V-FITC/propidium iodide (PI) double staining was carried out for the quantitative discrimination of apoptotic cells. Phosphatidylserine (PS) is a cell cycle signaling phospholipid located inner side of the membrane of a healthy cell but is reverted to the outer membrane for recognition by neighboring cells at the time of apoptosis. Hence, the translocation of phosphatidylserine is a morphological hallmark of apoptosis and can be spotted by its binding with fluorescently labeled annexin V which in turn detected by flow cytometry. Further the addition of PI to annexin V stained cells is used to discriminate and concomitantly quantify the live cells (lower left quadrant-annexin V(-)/PI(-)), early apoptotic cells (upper left quadrant-annexin V(+)/PI(-)) and late apoptotic cells (upper right-quadrant-annexin V(+)/PI(+)) using FACS. As projected in Figure 4(C), the incubation of Gd-PFH-NPs and C-Gd-PFH-NPs with C643 cells conspicuously induced apoptosis. It is worth to note that the titled complexes induce apoptosis even at very low concentrations which is less than their IC50. In comparison with control, the cell population was higher (6–9%) in annexin V(+)/PI(-) (upper left) quadrant indicating the induction of early apoptosis (Figure 4(E)). This effect was ascertained to be high for C-Gd-PFH-NPs than the Gd-PFH-NPs analogous with the results of MTT, and AO-EB staining assays. It is to note that the test samples displayed comparatively better apoptotic induction on C643 cells.

### 3.5. *In vitro* ultrasound imaging

**Figure 5.**
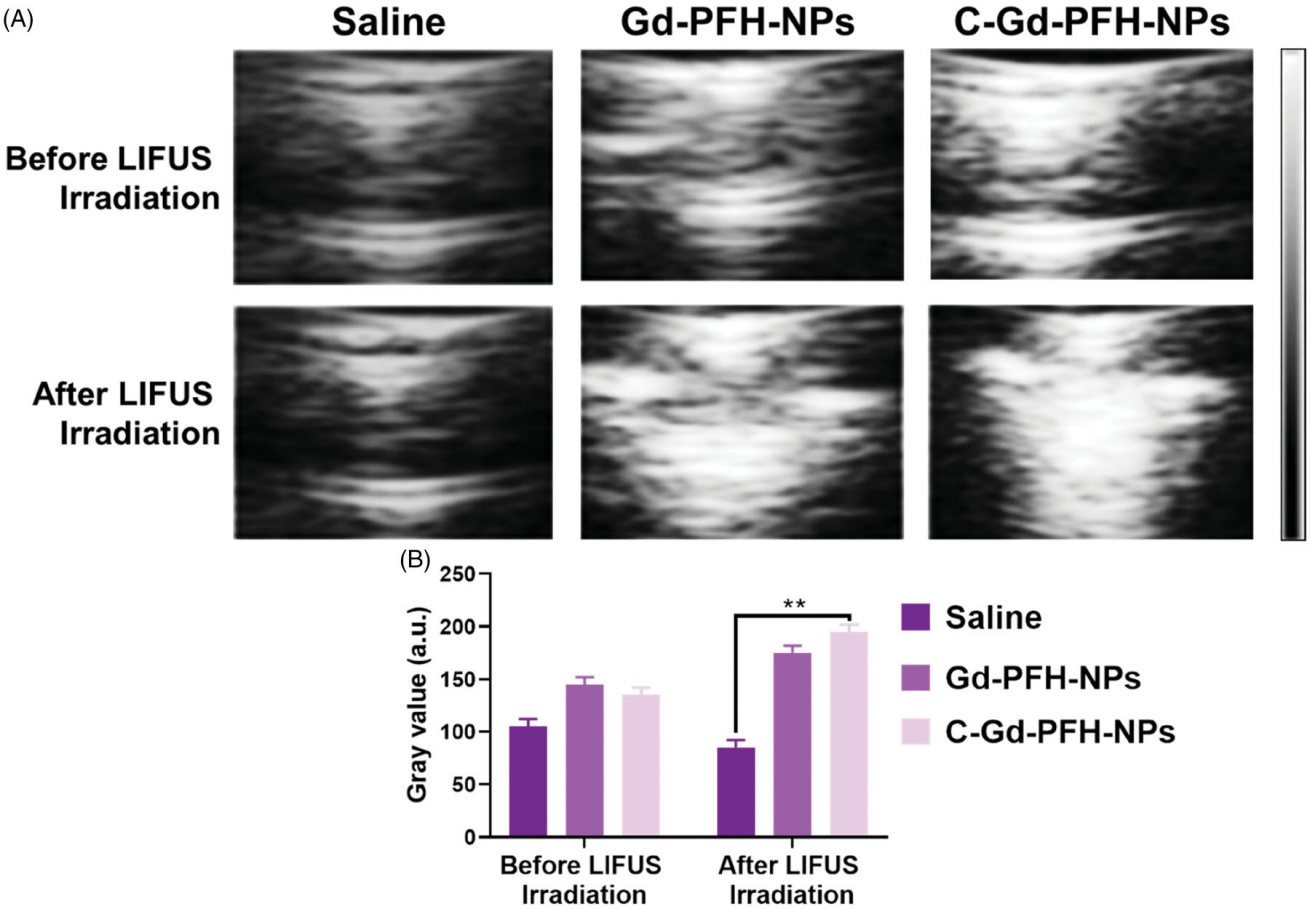
A) In vitro B-mode US imaging of Saline, Gd-PFH-NPs, and C-Gd-PFH-NPs before and after NIR laser irradiation. B) Gray values of B-mode US imaging of Saline, Gd-PFH-NPs, and C-Gd-PFH-NPs before and after NIR laser irradiation. **p < .01.

This outcome recommended that C225 eased the directing of tumor tissue accretion, and huge quantities of microbubbles were produced when phase-changing NAs were subjected to ADV at the LIFUS triggered tumor site, resultant in improved US imaging (Figure 5(A)). Though, owing to the absence of C225-mediated targeting capacity, the Gd-PFH-NAs and C-Gd-PFH-NAs inadequate ADV could not effectively improve ultrasound imaging. Furthermore, apparent enrichment without LIFUS irradiation was not found in the Gd-PFH-NAs and C-Gd-PFH-NAs alone could not *in vitro* improve the ultrasound imaging shown in Figure 5(B). These findings showed that because of their relative stability, Gd-PFH-NAs and C-Gd-PFH-NAs were appropriate as ultrasound imaging agents and efficient *in vivo* nanocarriers. The above information were compatible with the outcomes of ultrasonic imaging, additional checking the effectiveness of the beleaguered ultrasonic of C-Gd-PFH-NAs lower than LIFUS irradiation and local LIFUS radioactivity can boost the precision of phase changing C-Gd-PFH-NAs.

### 3.6. Histological evaluation for systemic toxicity

**Figure 6.**
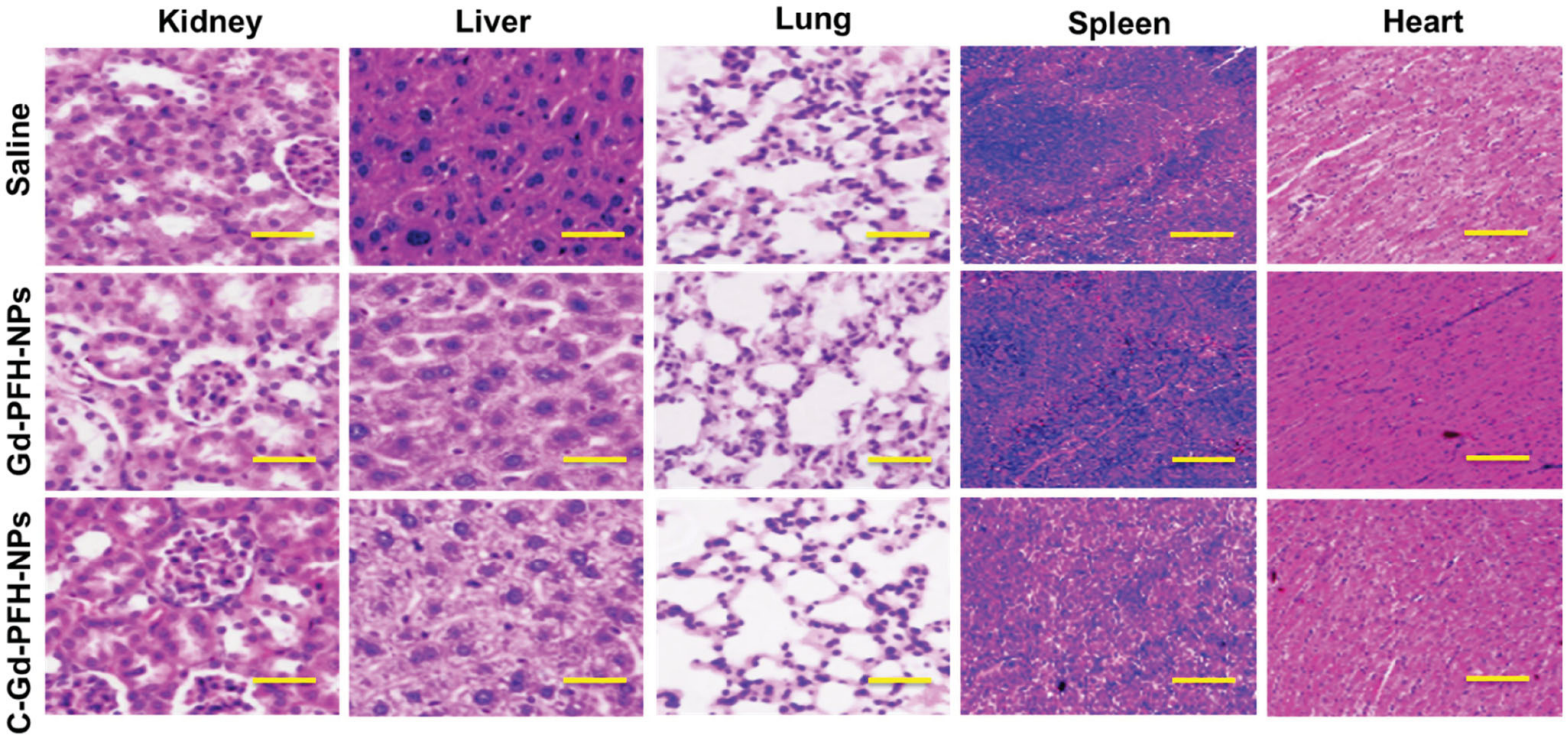
H&E staining of the major organs (kidney, liver, lung, spleen and heart) excised from different treatment mice groups. Scale bar: 100 μm.

The efficiency of anticancer chemotherapeutic drugs is mainly validated by its selective action toward cancer tissues leaving the normal organs undamaged. After the verification of low systemic toxicity in the mice injected with Gd-PFH-NPs (2.5, and 5 mg/kg), and C-Gd-PFH-NPs (2.5, and 5 mg/kg), histological analyses were carried out to identify the structural changes in the tissues of vital of organs inclusive of heart, liver, spleen, lung, and kidney of the mice treated with Gd-PFH-NPs and C-Gd-PFH-NPs and compared with control, the saline received mice. Figure 6 represented the histological sections of the heart, liver, spleen, lung, and kidney stained with hematoxylin and eosin (H&E).The photomicrographs of the liver and spleen of the control, Gd-PFH-NPs and C-Gd-PFH-NPs treated groups displayed normal cellular morphology. Under optical microscopy examination, the heart, lung, and kidney of Gd-PFH-NPs and C-Gd-PFH-NPs treated animals showed normal cardiac muscle fibers, normal alveolar, and normal glomerular histological characteristics respectively which were found to be similar histological architecture as those of the control group with no treatment-related inflammatory response.

### 3.7. *In vivo* antitumor efficacy in C643 xenograft tumor model

**Figure 7.**
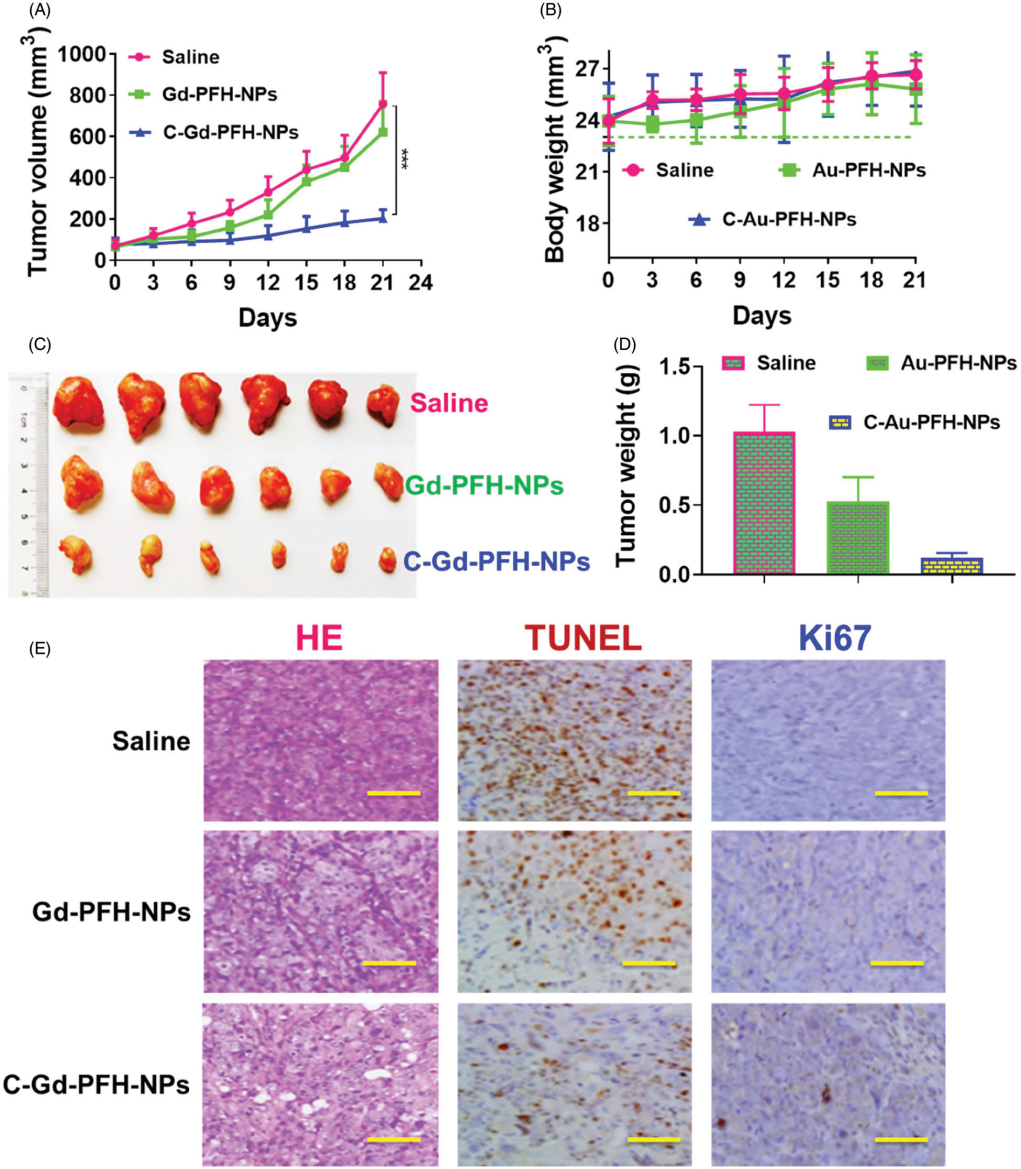
In vivo antitumor activity of Saline, Gd-PFH-NPs, and C-Gd-PFH-NPs compared to saline. C643 tumor xenograft-bearing BALB/c nude mice were administered with various drugs via intravenous injection at days 0, 3 and 6. A) Changes in tumor volumes. B) Body weights. C) Represent tumor photograph. D) Tumor weights. The data are presented as the means ± SD (n = 7). E) Representative H&E staining, Ki67, and TUNEL histopathological analysis of the tumors. ***p ≤ .005, compared to each normalized control.

To establish the potential of Gd-PFH-NAs and C-Gd-PFH-NAs to be translated to clinical applications, we assessed the antitumour efficacy *in vivo* was explored in subcutaneous C643 models, interested by the notable therapeutic efficacy of the mixture of Gd-PFH-NAs and C-Gd-PFH-NAs *in vitro*. In order to demonstrate the therapy impact, numerical data for separate groups of mice were drawn (Figure 7(A–C)). The therapeutic effectiveness was evaluated by tracking changes in each group’s tumor volume. The tumor in the saline groups the debauched and there was no significant decrease in the tumor dimensions in the C-Gd-PFH-NAs group, indicating that the dose of C-Gd-PFH-NAs was dependable *in vivo* and the well-known epidermal growth factor is target for tumor cell identification and treatment. These findings showed that C-Gd-PFH-NAs in nude mice could additional enhance the therapeutic effect of anaplastic subcutaneous thyroid cancer. Simultaneously, compared with control (saline) groups, H&E, procaspase 9 (brown), cleaved-caspase 3 (brown) expression levels are enhanced. Then, the Ki67 staining and TUNEL assay were showed to measure the apoptosis of the tumor *in vivo* (Figure 7(D)) Furthermore, during the course of therapy, C-Gd-PFH-NAs show no statistically important distinction in body weight between all mice groups. But, Gd-PFH-NAs significantly reduce body weight. The results of procaspase 9, cleaved caspase 3 histopathology analyses were consistent with the results of these therapeutic studies, showing extensive apoptosis and reduced cell proliferation caused by the C-Gd-PFH-NAs treatments (Figure 7(D)). The above findings obviously showed that in nude mice, the combination of C-Gd-PFH-NAs attained a notable excellent therapeutic effect counter to ATC, importance the security of beleaguered tumor treatment. This diagnostic approach is a preferred method for ATC of the thyroid, significantly improving the healing capacity lacking noticeable side effects.

## 4. Conclusion

The data presented here highlight a strategy and rationale for improving both the safety and effectiveness of Au-PFA-NAs. As the synthetic Au-PFA-NAs and C-Au-PFA-NAs are fully biocompatible composites with minimal modifications, the safety risks have been minimized when considering their clinical translation. Furthermore, given the ability of Au-PFA-NAs to overcome the cetuximab (C225)-conjugated C-Au-PFA-NAs, our approach is expected to have high value as an optional therapeutic platform for treating patients with drug-resistant cancer. Lastly, in addition to taxane agents, we envision that this C-Au-PFA-NAs-based approach could be a simple, yet broadly applicable strategy for improving cytotoxic nanotherapeutics with other antitumor agents.
